# A global assessment of urban extreme weather early warning systems and public health engagement

**DOI:** 10.2471/BLT.24.292205

**Published:** 2025-03-07

**Authors:** Mary Catherine Sheehan, Ana Boned-Ombuena, Lucinda Cash-Gibson, Alexa Damis-Wulff, Mary A Fox

**Affiliations:** a Department of Health Policy and Management, Johns Hopkins Bloomberg School of Public Health, 615 N Wolfe St, Baltimore, MD 21205, United States of America (USA).; b General Directorate for Public Health, Valencia, Spain.; c Barcelona School of Management, Universitat Pompeu Fabra, Barcelona, Spain.; d Oregon Health Authority, Salem, USA.

## Abstract

**Objective:**

To assess extreme weather early warning systems in large cities across the world.

**Methods:**

Among cities with populations above 1 million reporting to the Carbon Disclosure Project Cities Adaptation Actions database from 2021 to 2023, we included those providing a description of at least one adaptation action for a climate hazard in at least one year. We identified cities reporting early warning systems using the United Nations Early Warnings for All framework, which includes four pillars: risk knowledge, hazard monitoring and forecasting, warning communication and preparedness. We also tracked public health engagement in these systems.

**Findings:**

We identified 182 cities, of which 71 described full early warning systems across the four pillars. Cities in high- and upper middle-income countries described early warning systems nearly three times more often than those in low- and lower middle-income countries. Multihazard early warning systems  were reported by 35 (49%) cities, and many of these involved institutionalized cross-sectoral coordination and funded at least one activity from their own resources. Health was reported as a goal of early warning systems by 58 (82%) cities, although just 29 (41%) indicated a specific role for public health agencies.

**Conclusion:**

These findings suggest that many large cities are not covered by these health-protective systems. We recommend development of a city-specific framework for early warning systems that identifies roles for health, and scaling up of these tools, particularly in cities in low- and lower middle-income countries, to ensure strengthened adaptive urban resilience against climate threats.

## Résumé

**Objectif:**

Évaluer les systèmes d’alerte précoce en cas de phénomènes météorologiques extrêmes dans les grandes villes du monde entier.

**Méthodes:**

Parmi les villes qui comptent plus de 1 million d’habitants et qui ont annoncé des actions d’adaptation dans la base de données du Carbon Disclosure Project de 2021 à 2023, nous avons inclus celles qui ont fourni une description d’au moins une action d’adaptation à un risque climatique au cours d’au moins une année. Nous avons identifié les villes ayant mis en place des systèmes d’alerte précoce en nous basant sur le cadre du programme «Alertes précoces pour tous» des Nations unies, qui comprend quatre piliers: la connaissance des risques, la surveillance et la prévision des dangers, la communication des alertes et la préparation. Nous avons également fait le suivi de l’engagement du secteur de la santé publique au sein de ces systèmes.

**Résultats:**

Nous avons identifié 182 villes, dont 71 ont décrit des systèmes d’alerte précoce complets couvrant les quatre piliers. Les villes des pays à revenu élevé et intermédiaire de la tranche supérieure ont décrit des systèmes d’alerte précoce près de trois fois plus souvent que celles des pays à revenu faible et intermédiaire de la tranche inférieure. Des systèmes d’alerte précoce multirisques ont été signalés par 35 villes (49%), et nombre d’entre elles ont mis en place une coordination intersectorielle institutionnalisée et ont financé au moins une activité sur fonds propres. La santé figurait parmi les objectifs des systèmes d’alerte précoce de 58 villes (82%), bien que seules 29 (41%) aient attribué un rôle spécifique aux agences de santé publique.

**Conclusion:**

Ces résultats suggèrent qu’un nombre élevé de grandes villes ne sont pas couvertes par ces systèmes de protection de la santé. Nous recommandons l’élaboration d’un cadre spécifique aux villes pour les systèmes d’alerte précoce qui identifie les rôles de la santé, ainsi que le renforcement de ces outils, en particulier dans les villes des pays à faible revenu et à revenu intermédiaire de la tranche inférieure, afin de garantir le renforcement de la résilience adaptative des villes face aux menaces climatiques.

## Resumen

**Objetivo:**

Evaluar los sistemas de alerta temprana ante fenómenos meteorológicos extremos en grandes ciudades de todo el mundo.

**Métodos:**

Se incluyeron en el estudio las ciudades con más de un millón de habitantes que informaron al Carbon Disclosure Project sobre sus acciones de adaptación entre 2021 y 2023, y que describieron al menos una medida de adaptación para un peligro climático en al menos un año. Se identificaron las ciudades que informaron sobre la existencia de sistemas de alerta temprana conforme al marco Alertas tempranas para todos de las Naciones Unidas, que comprende cuatro pilares: conocimiento del riesgo, monitoreo y previsión de peligros, comunicación de alertas y preparación. Asimismo, se analizó la participación del sector sanitario en estos sistemas.

**Resultados:**

Se identificaron 182 ciudades, de las cuales 71 describieron sistemas de alerta temprana completos en los cuatro pilares. Las ciudades en países de ingresos altos y medianos altos informaron sobre la existencia de sistemas de alerta temprana casi tres veces más que aquellas en países de ingresos bajos y medianos bajos. Se documentó la presencia de sistemas de alerta temprana multirriesgo en 35 ciudades (49%), muchas de las cuales contaban con mecanismos institucionalizados de coordinación intersectorial y financiaban al menos una actividad con sus propios recursos. En 58 ciudades (82%), la salud se mencionó como un objetivo de los sistemas de alerta temprana, aunque solo 29 (41%) indicaron un papel específico para las entidades sanitarias.

**Conclusión:**

Estos hallazgos sugieren que muchas grandes ciudades no cuentan con estos sistemas de protección de la salud. Se recomienda desarrollar un marco específico para sistemas de alerta temprana en ciudades que defina los roles del sector sanitario y ampliar el alcance de estas herramientas, en particular en ciudades de países de ingresos bajos y medianos bajos, para fortalecer la resiliencia urbana adaptativa frente a las amenazas climáticas.

## ملخص

الغرض تقييم أنظمة الإنذار المبكر للطقس المتطرف في المدن الكبرى في جميع أنحاء العالم.

الطريقة من بين المدن التي يزيد عدد سكانها عن مليون نسمة، والتي تقوم بالإبلاغ إلى قاعدة بيانات "إجراءات التكيف للمدن التابعة لمشروع الكشف عن الكربون" من عام 2021 إلى عام 2023، قمنا بتضمين المدن التي قدمت وصفًا لإجراء واحد على الأقل من إجراءات التكيف مع المخاطر المناخية في عام واحد على الأقل. لقد قمنا بتحديد المدن التي أبلغت عن أنظمة الإنذار المبكر باستخدام إطار عمل "الإنذارات المبكرة للجميع من الأمم المتحدة"، والذي يتضمن أربعة محاور: معرفة المخاطر، ومراقبة المخاطر والتنبؤ بها، واتصالات التحذير والاستعداد. كما تتبعنا أيضًا المشاركة في مجال الصحة العامة في هذه الأنظمة.

النتائج قمنا بتحديد 182 مدينة، قامت 71 منها بوصف أنظمة الإنذار المبكر بالكامل عبر المحاور الأربعة. المدن من الدول ذات الدخل المرتفع، والدخل المتوسط العالي، قامت بوصف أنظمة الإنذار المبكر أكثر بثلاث مرات تقريبًا من تلك الموجودة في الدول ذات الدخل المنخفض، والدخل المتوسط المنخفض. وقد أبلغت 35 مدينة (%49) عن أنظمة الإنذار المبكر متعددة المخاطر، وقد اشتمل العديد منها على تنسيق مؤسسي عبر القطاعات، وقامت بتمويل نشاط واحد على الأقل من مواردها الخاصة. وأبلغت 58 مدينة (%82) أن الصحة هي أحد أهداف أنظمة الإنذار المبكر، على الرغم من أن 29 مدينة فقط (%41) أشارت إلى دور محدد للهيئات الصحية العامة.

الاستنتاج تشير هذه النتائج إلى أن العديد من المدن الكبرى لا تخضع للحماية بأنظمة الحماية الصحية هذه. نحن نوصي بتطوير إطار عمل خاص بكل مدينة لأنظمة الإنذار المبكر بحيث يحدد الأدوار الخاصة بالصحة، وتوسيع نطاق هذه الأدوات، وخاصة في المدن في الدول ذات الدخل المنخفض، والدخل المتوسط المنخفض، لضمان تعزيز المرونة الحضرية التكيفية ضد التهديدات المناخية.

## 摘要

**目的:**

评估全球大城市的极端天气预警系统。

**方法:**

在 2021 年至 2023 年期间向碳排放披露项目城市适应行动 (Carbon Disclosure Project Cities Adaptation Actions) 数据库提交了对应报告且人口超过 100 万的城市中，我们将那些针对至少 1 年内的气候灾害提交了至少一份适应气候变化行动方案的城市纳入了评估范围。我们采用联合国全民预警 (Early Warnings for All) 框架来确定报告了预警系统的城市，其中全民预警框架包括四大支柱，分别是风险知识、灾害监测与预报、预警传播和备灾。我们还在这些系统中追踪了公共卫生参与情况。

**结果:**

我们确定了 182 个城市，其中 71 个城市都从四大支柱各个方面对整个预警系统进行了描述。高收入和中等偏上收入国家的城市描述预警系统的频率几乎是低收入和中等偏下收入国家城市的三倍。35 个城市（占 49%）报告了多灾种预警系统，其中大多数城市都参与了有组织的跨部门协调并用自己的资源资助了至少一项活动。尽管只有 29 个城市（占 41%）表明公共卫生机构发挥了特定作用，但根据 58 个城市（占 82%）提交的报告，卫生防护才是预警系统的目标。

**结论:**

这些调查结果表明，许多大城市都不在此类卫生防护系统的覆盖范围内。我们建议针对不同城市专门制定相应的预警系统框架以确定卫生机构所发挥的作用，并建议对此类工具进行升级（尤其是在低收入和中等偏下收入国家的城市），以确保提高城市应对气候威胁时在适应气候变化方面的复原力。

## Резюме

**Цель:**

Оценить работу систем раннего предупреждения об экстремальных погодных условиях в крупных городах по всему миру.

**Методы:**

Среди городов с населением свыше 1 миллиона человек, которые прислали отчеты для базы данных «Действия городов в порядке адаптации Проекта по раскрытию информации о выбросах углерода» (Carbon Disclosure Project Cities Adaptation Actions) в период с 2021 по 2023 год, авторы включили в исследование те, у которых в отчете имелось описание по меньшей мере одного адаптационного действия относительно климатических опасных факторов в течение хотя бы одного года. Были идентифицированы города, сообщавшие о наличии систем раннего предупреждения об экстремальных погодных условиях, в которых использовался рамочный подход Объединенных Наций «Ранние предупреждения для всех» (Early Warnings for All), включающий четыре основополагающих принципа: знание рисков, мониторинг и прогнозирование опасных факторов, обмен предупреждениями и подготовленность. Также отслеживалась роль служб общественного здравоохранения в этих системах.

**Результаты:**

Были идентифицированы 182 города, из которых 71 предоставил описания полноценной системы раннего предупреждения, основанной на всех четырех принципах. В странах со средним и средне-высоким уровнем доходов населения города упоминали о наличии систем раннего предупреждения почти в три раза чаще, чем в странах с низким и средне-низким уровнем доходов. Системы раннего предупреждения, ориентированные на множественные виды угроз, имелись в 35 городах (49%), при этом многие из них подразумевали скоординированную работу учреждений из разных секторов и финансировали по меньшей мере один вид деятельности за счет собственных ресурсов. Сохранение здоровья было заявленной целью системы раннего предупреждения в 58 городах (82%), хотя всего лишь в 29 городах (41%) была указана конкретная роль органов общественного здравоохранения.

**Вывод:**

Эти результаты говорят о том, что многие крупные города не охвачены защитными системами здравоохранения. Авторы рекомендуют разработать специальный рабочий процесс конкретно для городов, который бы определял роль органов здравоохранения в системах раннего предупреждения и давал бы возможность масштабирования соответствующих средств осуществления, в частности, в странах с низким и средне-низким уровнями доходов населения, что позволит укрепить адаптивную устойчивость городов в отношении климатических угроз.

## Introduction

Large cities are particularly vulnerable to extreme weather events related to climate change because of their high population density, concentrated infrastructure networks and economic activity.^1^^,^^2^ Flooding, rainstorms, heat or drought are of concern for most cities,^3^ and many face risk from multiple hazards,^4^^,^^5^ which can lead to loss of lives, livelihoods and well-being.^6^ Risks are higher among disadvantaged groups, including poor, displaced and minority populations as well as children, women, older adults and those with pre-existing health conditions.^7^ The 3.4 billion people living in large cities of low- and middle-income countries are among the most exposed to extreme weather hazards,^8^ and the nearly 1 billion residents of informal settlements are particularly vulnerable because of poor housing, overcrowding and lack of services.^9^ Maximizing strategies to protect the health of urban populations most at risk from extreme weather disasters is both a challenge and an opportunity to reduce inequities as the climate changes.

One promising strategy is extreme-weather hazard early warning systems, which issue alerts based on weather forecasts and provide guidance for anticipatory actions. Early warning systems have been shown to protect lives, livelihoods and assets at relatively low cost,^6^^,^^8^^,^^10^ making them a valued health-supportive policy tool.^11^^,^^12^ Because of these benefits, early warning systems have been promoted under the Sendai Framework for Disaster Risk Reduction, which monitors disaster mortality, morbidity and displacement;^13^ and via climate adaptation recommendations of the Intergovernmental Panel on Climate Change and others.^1^^,^^2^^,^^7^^,^^14^^–^^16^ According to the World Meteorological Organization (WMO), early warning systems rely on four functioning pillars: (i) hazard and risk knowledge; (ii) monitoring and forecasting; (iii) warning communication; and (iv) preparedness.^17^ Key challenges to system effectiveness include coordination across these pillars and their responsible agencies, and ensuring community engagement.^18^

Although early warning systems are often implemented at national or regional level, many cities have also developed complementary early warning capacities. Urban early warning systems can help improve targeting and communication by using knowledge of local hazard patterns and proximity to at-risk populations.^19^ However, the effectiveness of urban early warning systems depends on coordinated governance, both to integrate functions provided at higher level (for example, climate and weather forecasting services) and across city agencies (for example, emergency management, urban planning and public health).^20^ Public health agencies, in particular, have useful tools for early warning, including health surveillance, risk assessment, and relationships with emergency management teams and hospitals.^7^^,^^21^^–^^23^ Co-creation of protocols with local communities is also essential to ensure equitable and understandable warnings.^18^^,^^24^

Recognition that climate-related extreme weather may involve more than one hazard has led to expansion of early warning systems to include multiple hazards. These multihazard early warning systems are integrated alerts that inform of multiple imminent hazard events simultaneously, cumulatively or in cascading fashion.^25^ To improve countries’ capacities to protect people from such events, the United Nations (UN) launched the Early Warnings for All initiative in 2022.^17^ The initiative has a dashboard, which monitors national-level early warning systems worldwide, and 101 countries confirm having some national multihazard early warning system capacity.^26^ However, little is known about city early warning system capacity, and a third of the world’s population is thought not to be covered, particularly those in highly urbanized low- and lower middle-income countries.^26^ To better understand the urban multihazard early warning coverage gap, and the role of health in these systems, we aimed to assess extreme weather early warning systems in large cities worldwide. 

## Methods

We screened descriptions of climate adaptation actions in a convenience sample of large cities reporting to the Carbon Disclosure Project Cities Adaptation Actions database. This database is the most comprehensive publicly-available international source for city self-report adaptation initiatives, and it forms the reporting framework for several international city networks.^27^ Updated annually by city staff following standardized guidance, the database is used for sharing city practices and supporting scholarly research.^6^^,^^28^

We downloaded city data for 2021–2023 to an Excel spreadsheet (Microsoft, Redmond, United States of America) via the Carbon Disclosure Project Open Data Portal.^27^ Eligibility criteria for inclusion were having a population over 1 million and providing a description of at least one adaptation action for a climate hazard in at least one of the study years. For each eligible city, we extracted and coded eight variables: (i) population; (ii) climate hazard; (iii) climate action category; (iv) climate adaptation action description (from city-reported written summaries); (v) health sector involvement; (vi) perceived health co-benefits; (vii) funding sources; and (viii) implementation status.

To group eligible cities based on the presence of an early warning system, we established criteria using the WMO’s four-pillar structure. Initial criteria were refined through piloting in a group of cities (online repository).^29^ We analysed the content of adaptation action descriptions and assessed their fit within these criteria using keywords. A city was considered to have an integrated multihazard early warning system capacity when it reported adaptation actions addressing more than one hazard in each of the four pillar areas; and a single-hazard early warning system capacity when it reported actions addressing one hazard across the four pillars. Actions contributing to early warning systems but not including all pillars were also tracked.

For each action we tabulated reported health sector engagement, health co-benefits, funding source and implementation status. By reviewing action descriptions, we identified whether cities reported health as an early warning goal and assessed any described role for the local public health agency in early warning systems-related actions. We further assessed whether actions were targeted to at-risk populations, and any formalized coordinating governance arrangements. One author reviewed the reporting of city adaptation actions first, and the remaining authors each independently reviewed a sample of city action descriptions. Any inconsistencies were resolved by consensus. Reporting of our study follows the *Strengthening the reporting of observational studies in epidemiology* checklist.^30^

## Results

Of over 570 cities reporting during the study period, 182 met the inclusion criteria; 128 (70%) were located in high- or upper middle-income countries, and 54 (30%) in low- or lower middle-income countries. While all World Health Organization (WHO) regions were represented, only four eligible cites were located in the Eastern Mediterranean Region (Table 1). 

**Table 1:** Characteristics of eligible cities assessed as having an extreme weather early warning system, as reported to the Carbon Disclosure Project, 2021–2023

Characteristic	No. of cities (%)			
	Eligible (*n* = 182)	Early warning system		
		Any system (*n* = 71)	Single-hazard system (*n* = 36)	Multihazard systems (*n* = 35)
**WHO Region**				
African	31 (17)	4 (6)	4 (11)	0 (0)
Americas	63 (35)	31 (44)	16 (44)	15 (43)
South-East Asia	22 (12)	4 (6)	4 (11)	0 (0)
European	26 (14)	14 (20)	5 (14)	9 (26)
Eastern Mediterranean	4 (2)	0 (0)	0 (0)	0 (0)
Western Pacific	36 (20)	18 (25)	7 (19)	11 (31)
**World Bank country income category**				
High income	61 (34)	32 (45)	9 (25)	23 (66)
Upper middle-income	67 (37)	30 (42)	19 (53)	11 (31)
Lower middle-income	43 (24)	8 (11)	7 (19)	1 (3)
Low income	11 (6)	1 (1)	1 (3)	0 (0)

WHO: World Health Organization.

Note: Eligible cities had a population of over 1 million and provided a description in Carbon Disclosure Project Open Data Portal^27^ of at least one adaptation action for a climate hazard in at least one of the study years.

**Fig. 1:**
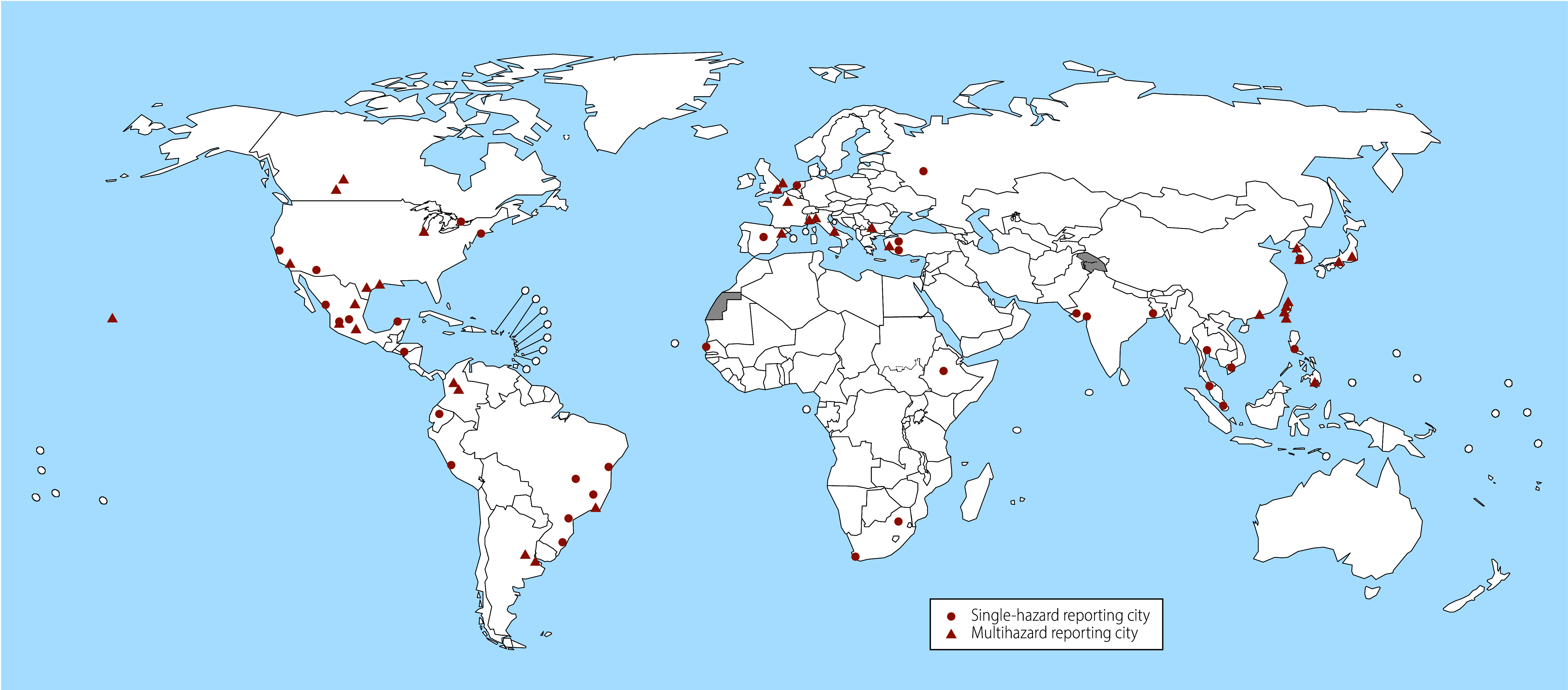
Large cities reporting extreme weather early warning systems to the Carbon Disclosure Project, 2021–2023

Among the 182 eligible cities, 71 (39%) reported actions in all four early warning systems pillars for one or more climate-related hazard and were included in the early warning systems group. In terms of geographic coverage, cities in the high- and upper middle-income countries of the Americas, European and Western Pacific regions combined (constituting 70% of eligible cities) represented 87% (62/71) of the cities in the group (Fig. 1). Half of eligible cities reported urban early warning systems activities across all pillars in the Americas (31/63), European (14/26) and Western Pacific (18/36) regions compared with 13% (4/31) of cities in the African Region, 18% (4/22) in the South-East Asia Region and none of the four cities in the Eastern Mediterranean Region (Table 1). 

The primary hazards associated with city early warning efforts were flooding (43 cities), followed by extreme heat (39 cities). More cities in the South-East Asia and Western Pacific regions reported having a warning system for flooding, while warning systems in cities of the Americas, African and European regions were divided between flooding and extreme heat. 

Thirty-nine cities (21%) reported actions in more than one but less than four early warning systems pillars. The pillars most often unreported were monitoring and forecasting (pillar 2) and warning communication (pillar 3). Seventy-two cities (40%) reported no or one early warning systems-linked action, which was often preparedness (pillar 4); half were in lower middle-income countries and low-income countries in the African and South-East Asia regions. 

We next examined whether early warning systems focused on single or multihazards. Of the 71 cities with early warning systems, 36 (51%) reported single-hazard systems, while 35 (49%) reported multihazard systems (Fig. 1; Table 1). Nearly all (34/35) cities with multihazard early warning system capacity were in high- and upper middle-income countries. Multihazard systems covered an average of 3 (range 2–7) extreme weather hazards per city, principally flooding (28 cities; 80%), followed by extreme heat (25 cities; 71%); and 19 (54%) cities reported both hazards. Other urban hazards reported in cities with multihazard system capacity were rainstorm, cyclone, drought, landslide, wildfire, cold, vector-borne disease, wind and dust. Several cities also reported early warning systems for earthquakes, volcanic eruptions and epidemics, as well as for other non-climate hazards such as traffic management and poisoning. Nearly half (17/35) of cities with multihazard systems reported cross-sectoral governance arrangements designed to support multihazard goals, ranging from legally constituted agencies for disaster warning coordination to combined hazard alert systems and multihazard risk atlases. Notably, 33 (95%) cities reporting multihazard systems funded at least one activity from their own resources. The majority (28/36; 78%) of cities with single-hazard early warning systems were also located in high- and upper middle-income countries. The most common single hazard was flooding (15/36; 42%), followed by extreme heat (14/36; 39%). Several of these cities also reported some collaborative governance measures. The majority of these cities (25/36; 70%) funded at least one activity from own resources, while other sources of funding included national or regional funds and international assistance.

**Fig. 2:**
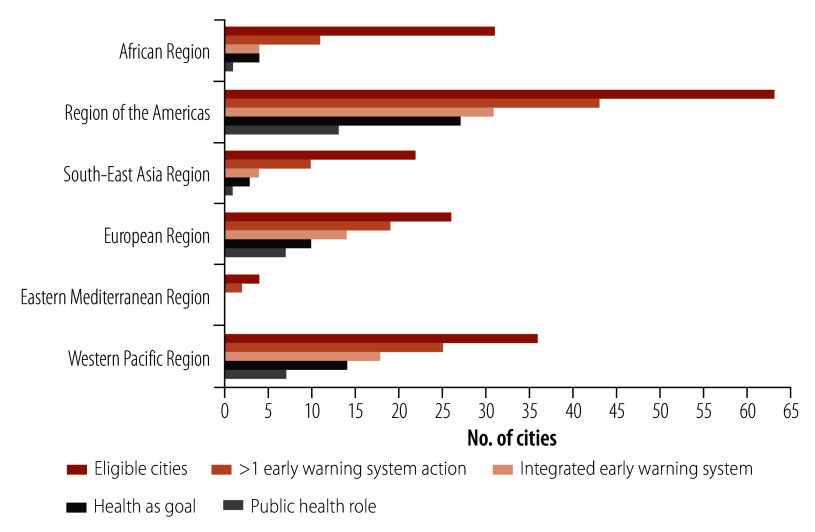
Number of cities reporting early warning systems actions and public health engagement parameters to the Carbon Disclosure Project, by WHO Region, 2021–2023

Most (58) cities with early warning systems had the goal of protecting human health for at least one hazard-specific early warning activity (Table 2 and Fig. 2). This action was characterized with wording such as “avoiding loss of lives,” “reducing impacts on vulnerable populations” and “protecting communities.” Health co-benefits from early warning actions were reported by 53 cities (75%), and health was reported as a sector engaged in early warning actions by 47 cities (66%). A role for local public health agencies was reported by 29 cities (41%), more frequently for cities with multihazard early warning systems and in the Americas, European and Western Pacific regions. Extreme heat was the hazard for which the highest proportion of cities (22/29; 76%) reported health engagement in early warning systems, followed by vector-borne disease. Targeting of early warning system activities to at-risk groups was reported by 35 cities: two thirds (23) of those reporting multihazard early warning systems, and one third (12) of those reporting single-hazard early warning systems. The most frequently-cited risk groups were older adults and children in association with heat, those exposed to flooding (for example, living on steep slopes or low areas), and people with low income, who were homeless or living in inadequate housing. Twelve cities reported formal coordination involving public health; these included public health partnerships with national weather services and universities for monitoring and forecasting (pillar 2), and as part of centralized city agencies for warning communication (pillar 3). Such coordination was more common in multihazard than single-hazard early warning system-reporting cities. Box 1 shows examples of early warning activities with health engagement by WMO pillar.

**Table 2:** Health engagement parameters in cities reporting early warning systems actions in all four pillars to the Carbon Disclosure Project, by hazard, region and country income category, 2021–2023

Characteristic	Total (*n* = 71)	No. cities (%)			
		Health co-benefit (*n* = 53)	Health sector (*n* = 47)	Public health role (*n* = 29)	Health as goal (*n* = 58)
**Type of hazard**					
Multiple hazard early warning systems	35	31 (89)	28 (80)	20 (57)	32 (91)
Single hazard early warning systems	36	22 (61)	19 (53)	9 (25)	26 (72)
**WHO Region**					
African	4	4 (100)	2 (50)	1 (25)	3 (75)
Americas	31	22 (71)	20 (65)	13 (42)	27 (87)
South-East Asia	4	3 (75)	0 (0)	1 (25)	2 (50)
European	14	11 (79)	8 (57)	7 (50)	10 (71)
Eastern Mediterranean	0	NA	NA	NA	NA
Western Pacific	18	13 (72)	17 (94)	7 (39)	14 (78)
**World Bank country income category**					
High income	32	28 (88)	27 (84)	18 (56)	28 (88)
Upper middle-income	30	19 (63)	15 (50)	9 (30)	23 (77)
Lower middle-income	8	6 (75)	5 (63)	2 (25)	7 (88)
Low income	1	1 (100)	0 (0)	0 (0)	1 (100)

NA: not applicable; WHO: World Health Organization.

WHO: World Health Organization.

Box 1 **Examples of health role described in city early warning activities reported to the Carbon Disclosure Project, by early warning systems pillar**

***Multipillar***

*Centralized city early warning coordinating organizations*. The multihazard early warning system of Taipei, Taiwan, China, is coordinated across public health, networked infrastructure, social services and city administration, and it provides alerts for floods, dam failures, windstorms, fires, earthquakes, volcanoes, explosions and poisoning. Fifteen other cities, including Mexico City, Mexico, and Rio de Janeiro, Brazil, reported similar horizontally integrated multihazard early warning system capacity; many of these warnings are explicitly targeted to vulnerable groups.

*Heat-health action plans*. The city of Phoenix, United States of America, has developed a heat plan and an office and officer for heat response, with priorities such as heat awareness for outdoor spaces, homes, workplaces and schools. The city has hired a public health advisor to ensure integration of health into the city’s climate plans. Most other cities reporting early warning system activities for heat cited some form of health engagement, with targeting often focused on older, very young, poorly housed and isolated populations.

***Pillar 1. Risk knowledge***

In Rosario, Argentina, the public health agency maps extreme heat and zoonotic disease risk, and makes online climate and health epidemiological data publicly available to build awareness and foster community participation in tracking health determinants. Barcelona, Spain, maps heat hazard and health risk and incorporates it in the city’s climate adaptation plan. Several other cities reported a public health role in risk and vulnerability assessment and mapping and in the integration of these findings into city climate planning.

***Pillar 2. Monitoring and forecasting***

A partnership between health authorities and the national meteorological institute in Zapopan, Mexico, monitors weather conditions and provides early warning of vector-borne disease outbreaks; multiple other cities reported such efforts. In Quezon City, Philippines, water quality and climate parameters are monitored in collaboration with the public health agency to develop predictive models and anticipate waterborne outbreaks. Medellín, Colombia, and Rosario, Argentina, reported similar activities.

***Pillar 3. Warning and communication***

In Izmir, Türkiye, public health authorities have initiated a programme for climate change and health awareness targeted at those most at risk, including older people and those ill or disabled, poorly housed or with low income, with the goal of codeveloping effective strategies and capacity for household preparedness with the community. Several other cities reported health engagement with awareness raising, particularly for heat and flooding.

***Pillar 4. Preparedness***

The health authority in Can Tho City, Viet Nam, conducts cyclone-related drowning prevention for children and preparedness training for health workers. Denizli, Türkiye, has initiated enhanced hospital preparedness for heat-related illnesses, and increased collaboration with emergency services. Buenos Aires, Argentina, reported integrating informal settlements via housing regularization, and health and social programmes as part of flood hazard preparedness. Several cities reported similar preparedness programmes with health engagement.

## Discussion

In this assessment of 182 large cities reporting to an international climate adaptation database, 71 cities described activities addressing all four WMO early warning pillars. The majority of these cities were in high- and upper middle-income countries. A further 39 cities reported early warning activities in two or three of the four pillars, often covering multiple hazards, suggesting the potential to scale up these systems in these cities. However, the remaining cities did not report any early warning activity or reported mainly limited preparedness efforts; the majority of these cities were located in lower middle- and low-income countries. These findings suggest a concerning gap in urban early warning coverage for city dwellers in lower middle- and low-income countries, many of whom face some of the highest risk from extreme weather-related hazards.

Identified urban early warning systems were evenly split between single- and multihazard systems. A multihazard approach is especially relevant in the complex urban built environment.^31^ For example, in partnership with WMO, Shanghai, China, has pioneered a widely referenced multihazard early warning system addressing extreme heat, electric power outages, traffic safety, bacterial food poisoning and other hazards.^32^ WMO has also prioritized developing integrated urban services for climate.^33^ Yet, published case studies and reviews for urban early warning systems have most often focused on single hazards, including heat,^34^ flooding and landslides,^35^^,^^36^ cyclones^37^ and arbovirus outbreaks.^38^ Few articles report on multihazard early warning systems in cities or across cities, or on the role of the health sector in these systems in the urban context.^39^ We found that over half of cities with identified multihazard early warning systems addressed both flooding and extreme heat (with other common hazards including cyclones, landslides and wildfire), and that cities with multihazard early warning systems frequently reported good-practice early warning features. These features included targeting at-risk populations; cross-sectoral local governance structures; formalized partnerships with national or regional weather forecasting and other relevant agencies; community participatory initiatives; as well as recognition of health benefits, engagement of public health agencies and self-financing of activities. Such features were less frequently reported in cities with single-hazard early warning systems. Of concern, no cities in low-income countries and only one city in a lower-middle-income country reported a multihazard early warning system. Further studies are needed to identify best-practice features of multihazard early warning systems in large cities, including innovations in ways to scale up and increase coverage of these systems.

Most early warning systems-reporting cities considered population health a goal. However, only 29 cities reporting early warning systems described specific roles for public health agencies. Our findings align with previous research suggesting that public health departments are often on the sidelines of city climate adaptation,^40^ and are not fully included in disaster risk reduction and preparedness efforts. Yet, public health has been cited for its potential to enhance integration between disaster risk reduction and climate adaption via strategies such as early warning systems which are tools common to both efforts.^41^ Among extreme-weather early warning systems, heat-health plans have seen the highest level of health sector engagement;^42^^–^^44^ these heat-health plans showcase the need for iterative reassessments of early warnings as climate conditions change.^45^ With growing concern over the urban heat island effect, city uptake of such plans is strong, and local public health agencies often play leadership or supporting roles.^40^ For example, a study of city heat-health plans in the United States found public health agencies to be involved in all 21 plans surveyed, although these agencies led or co-led such plans less than one third of the time.^46^ Our study confirms these findings: a greater proportion of cities reporting health engagement parameters targeting heat compared to other hazards, and several cities noted that public health agencies led these activities. Researchers have hypothesized that heat-health plans may be a gateway for broader health engagement in local climate adaptation plans, including early warning for other hazards.^39^ The present study supports this hypothesis. Research also shows that in partnership with meteorological agencies, the public health sector has worked to shift climate-sensitive disease surveillance towards proactive outbreak forecasting.^7^^,^^47^^,^^48^ We observed evidence of this engagement in the reported monitoring of vector- and waterborne disease by several cities.

The WHO Climate Resilient Health Systems Operational Framework identifies measurable outputs and indicators for early warning systems, which include integrated disease surveillance and early warning, monitoring and progress tracking, communication and preparedness.^7^ These outcome areas are broadly consistent with the four-part WMO early warning systems pillar structure, although the two frameworks are not explicitly aligned. In assessing reported activities by early warning systems pillar, we found examples of health engagement consistent with many of these WHO outcome areas. 

Comparing our city-level results to a recent best-practice assessment of national early warning systems highlights both commonalities and difference.^18^ Similar to our findings, national-level monitoring in higher-income countries more frequently included hazard and risk mapping and vulnerability assessments (pillar 1) than in lower-income countries. As with national-level monitoring, cities also generally reported limited detail on protocols and partners for climate services (pillar 2). In contrast, while our city results suggested prioritization of outreach, emergency drills, resilience hubs and other community-focused efforts (pillars 3 and 4), these efforts were listed as gap areas at the national level. This finding suggests the potential important role of cities in ensuring effective communication to people at risk. Meanwhile, a national programme review of the Early Warnings for All initiative found that hazard monitoring and forecasting (pillar 2) and warning communication (pillar 3) activities were the most frequently reported, whereas risk knowledge (pillar 1) and preparedness (pillar 4) were the least frequently reported at national level.^12^ In contrast, we found a reverse pattern for city-reported adaptation actions. This finding suggests cities may be better equipped to address local risk knowledge and preparedness efforts than higher governance levels, which may warrant further study.

This study has limitations. Our sample is not representative of the world’s large cities, because the sample is based on voluntary reports from cities engaged in international climate and sustainability networks. While these networks have grown globally, the Carbon Disclosure Project database covers only a third of large cities with populations over 1 million.^49^ Furthermore, cities that did not report early warning activities to the database during the study years were not captured. While eligible cities covered half or more of large cities in some regions, other regions were poorly represented. In particular, few Eastern Mediterranean cities met our population size and reporting criteria. Additionally, while China accounts for an important share of the world’s large cities,^49^ few of these cities reported to Carbon Disclosure Project in 2021–2023. In addition, our data relied on self-reported activities over 3 years; making the reports susceptible to errors, biases and incomplete reporting of relevant early warning systems actions. Moreover, the Carbon Disclosure Project database is designed to report adaptation rather than early warning systems actions, and covers few health-related parameters. Finally, information on national and regional early warning systems activities, with which many city early warning systems activities are interconnected, was also not comprehensibly available in this data set. Therefore, the actual number of cities with early warning systems, and number of early warning systems actions, is likely to be higher.

While these limitations compromise the generalizability of the findings, this study aims to provide a complement to disaster risk reduction tracking, to suggest policy and practice considerations and to highlight research needs. We suggest that to improve protection for urban residents from extreme weather hazards, policy-makers should ensure that early warning systems are comprehensive, both in terms of activities conducted and hazards covered. Furthermore, strengthening cross-sectoral collaboration, including engagement of public health agencies, could enhance risk targeting, forecasting, communication and preparedness efforts. In high-risk, lower-income country cities, increased funding and support from international organizations and funding agencies may facilitate the expansion of urban multihazard early warning systems. Developing a city-specific framework to track and improve early warning activities, while aligning with WMO and WHO initiatives, could help further integrate the public health sector in early warning activities. Finally, further research is needed to identify best practices for scaling up urban multihazard early warning systems (Box 2).

Box 2 **Recommendations for extreme urban weather early warning systems**

***Local city policy and practice***

- Scale up city activities to cover all four early warning systems pillars, including complementing existing local, national or regional programmes; and scale up from single-hazard efforts (e.g. heat readiness plans) to multihazard systems that include other relevant city hazards, based on examples of evolving good practice urban multiple-hazard early warning systems such as those in Rio de Janeiro and Taipei (Box 1).
- Strengthen city cross-sectoral management of early warning efforts, including increased engagement of city public health agencies, to support improvements across pillars, including at-risk targeting (pillar 1), impact-based forecasts (pillar 2), improved local communication with difficult-to-reach populations (pillar 3), and community-focused training and drills (pillar 4); Box 1 identifies some promising practices.
- Expand partnerships between meteorological agencies and city public health authorities to strengthen monitoring and forecasting efforts (pillar 2), aiming to develop predictive models for disease outbreaks and health impact-based weather forecasts.

***Global policy and practice***

- Scale up funding and support for urban multihazard early warning systems (e.g. within the Early Warnings for All initiative) targeted to populous high-risk cities in lower-income countries with no or little early warning systems-related activity.
- Develop a city-specific framework and dashboard for population health-focused multihazard early warning systems; and enhance tracking of city early warning system activities, particularly in regions with few city reports.
- Align outcomes from the WMO Early Warnings for All initiative pillars and the WHO Climate Resilient Health Systems Operational Framework, to help ensure public health plays a strong role in early warning systems, particularly in connecting agencies, pillars and communities.

***Research***

- Evaluate urban multihazard early warning system-linked activities, including aspects such as targeting actions to at-risk populations, collaborative governance arrangements, and public health role across all four pillars to identify promising practices and pathways for scaling up to full multihazard early warning systems.

In conclusion, many large cities are poorly protected by early warning systems, particularly cities most vulnerable and exposed to climate-related extreme hazards. Actions are needed to scale up early warning systems in the large cities of low- and lower middle-income countries, in coordination with ongoing national efforts and the Early Warnings for All initiative. We also identified elements of good practice, which city policy-makers can pursue. At the international level, development of a city-specific framework for multihazard early warning systems with a health focus is needed. Most urgently, these tools and public health engagement with them, should be further developed in cities of lower-income countries, to ensure strengthened adaptive urban resilience against climate threats.
